# Evaluation of the offset static rope evacuation procedure: insights from a safe job analysis

**DOI:** 10.1186/s13049-024-01186-1

**Published:** 2024-03-18

**Authors:** Eirik Bjorheim Abrahamsen, Håvard Mattingsdal, Håkon Bjorheim Abrahamsen

**Affiliations:** 1 Department of Safety, Economics and Planning, University of Stavanger, 4036 Stavanger, Norway; 2 Rescue Technical Department, Norwegian Air Ambulance, 0182 Oslo, Norway; 3 Department of Anaesthesiology and Intensive Care, Stavanger University Hospital, 4011 Stavanger, Norway

**Keywords:** Safe job analysis, Risk, Risk analysis, Static rope evacuation, Offset technique, Helicopter emergency medical services, Rescue operations

## Abstract

**Background:**

Recently, the Norwegian Helicopter Emergency Medical Service (HEMS) has developed a procedure for a special type of static rope rescue operation, referred to as the offset technique. In this technique, the helicopter is offset from the accident site, and the HEMS technical crew member uses an offset throw line to gain access to the scene. Today, there is little practical experience of such operations, and a need has been identified for more knowledge on the potential hazards encountered during this type of operation. Such knowledge is of importance for further development of the procedure for the offset technique.

**Objective:**

To identify potential hazards for helicopter rescue operations using the static rope offset technique and, thereby, to improve the procedure for such operations. This may lead to improved safety for patients and crew members during offset rescue operations.

**Method:**

A Safe Job Analysis was used to identify the hazards of offset rescue operations. Such operations are divided into tasks and sub-tasks. For each sub-task, we identified potential hazards and suggested ways of preventing these.

**Results:**

Through the Safe Job Analysis, we suggest some changes in the existing procedure for the offset technique, to make it more robust against potential hazards.

**Conclusion:**

We have demonstrated the value of Safe Job Analysis for improving the static rope offset evacuation procedure. Our analysis has led to some changes in the procedure for offset rescue operations. This is the importance of having two throw lines and focusing on “why” in the procedure.

## Background

The time from an incident to the start of pre-hospital care is considered an influential factor determining patient outcome [1–4], particularly for severely injured or ill patients [1, 5–7]. Human External Cargo (HEC) operations is a small part of the Norwegian Helicopter Emergency Medical Services (HEMS) mission profile, but patients which need an extraction with HEC often either have no other alternatives or more time-consuming methods are utilized, e.g. ground based rescues. With reference to this, Norwegian HEMS is focusing on the development of new rescue operation techniques so that, in some situations, patient treatment can be started earlier than is currently the case.

As of today, Norwegian HEMS only use static rope evacuation. In a static rope rescue operation, the HEMS technical crew member (HCM) acts as the rescuer and is transported, underslung, to the accident site by a fixed rope. The length of the rope used differs between 10 and 60 m and depends on the actual situation (terrain) [1, 8]. During the static rope rescue operation, the pilot cannot see what is underneath the helicopter and is assisted in this phase by the emergency physician, who verbally communicates vertical and horizontal guidance. The HCM communicates with the other crew members by both a wireless intercom and standardized hand signals. After the HCM gains ground contact, he or she can disconnect from the static rope. The helicopter then leaves the scene with the static rope underneath it and normally hovers not far from and in sight of the scene of the accident [7, 8]. The helicopter returns to the scene following communication with the HCM after necessary patient treatment and preparations have been carried out. When the helicopter arrives, the HCM reconnects himself and the patient to the static rope, so that both can be evacuated, underslung, to the rig site before further patient treatment can be provided. The patient is then taken to the hospital by either helicopter or ambulance. An additional safety measure, which has shown its safety relevance for static rope missions in complex terrain, is the so-called double attachment procedure [8]. This method ensures that the HCM and patient are attached to a safety barrier at all times of the operation due to a high risk severity on site.

**Fig. 1:**
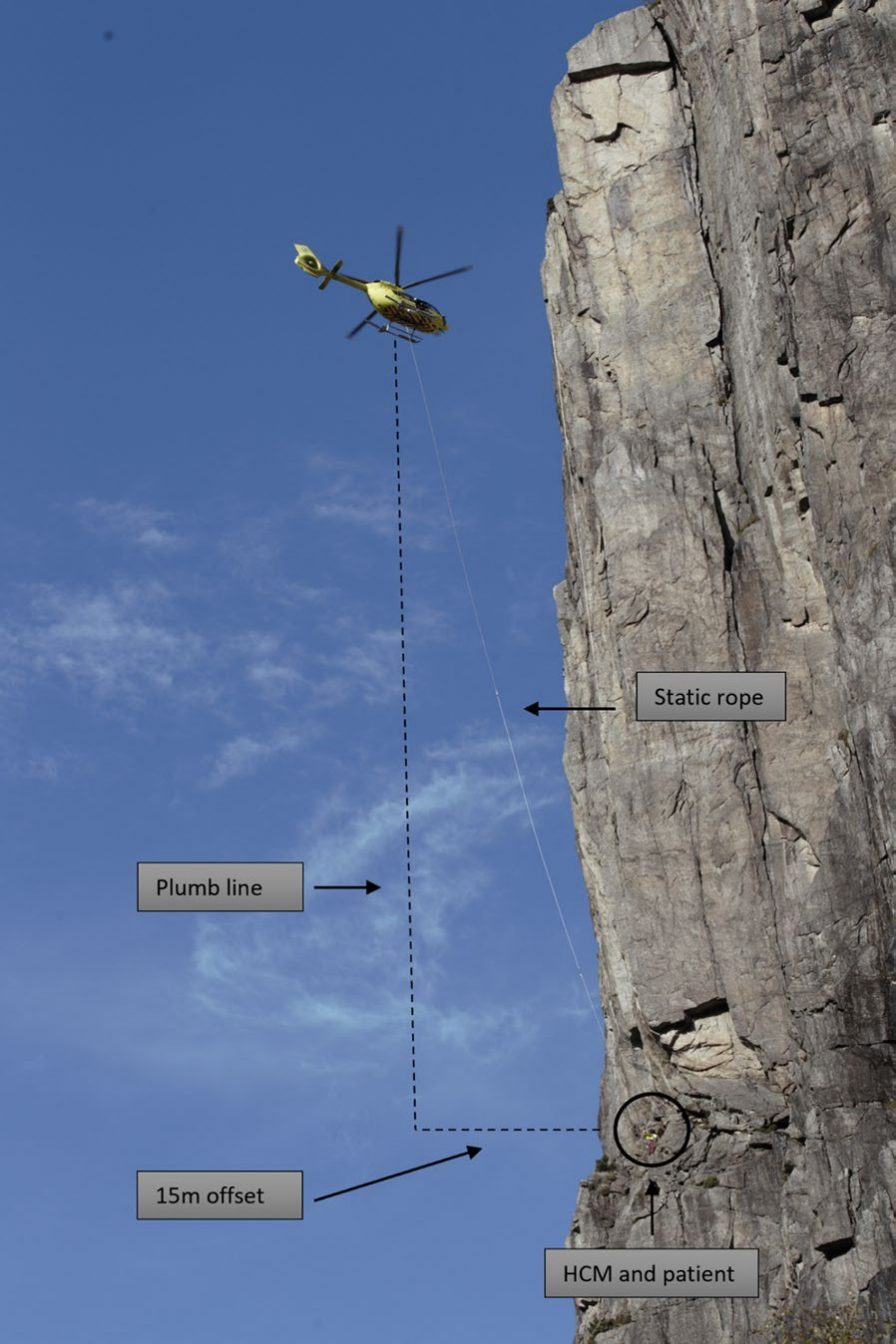
Helicopter is positioned out of plumb in an offset technique training mission. An evacuation from such a scene on a vertical wall with a controlled pendulum is considered safe due to there being no obstacles in the evacuation axis. Photo: Hugo Bergsaker

By focusing on improvements, the Norwegian service has identified situations in which the helicopter crew is unable to establish contact with the patient through traditional HEC methods, either hoist- or static rope rescue operations. In most cases, the helicopter lands at the scene of the accident, but, in situations where the helicopter cannot land close to the scene due to either forest- or mountainous terrain, a HEC method can be used. In some circumstances, however, it is not possible to achieve contact with the patient, even with the use of either a hoist or static rope. This is in situations where the helicopter cannot be positioned vertical above the accident site due to the terrain, e.g. a vertical or overhanging mountain wall (Fig. 1). These missions are rare, approximately 1–2 missions annually according to the Norwegian Alpine Rescue Groups (September 2023). As of today, there isn`t a national system for registering these missions. Even though the patient numbers are low, the potential health benefit for these patients could be substantial. It is with reference to this that a so-called offset technique has been developed, where the helicopter is out of plumb from the scene but where one can still make contact with the scene of the accident by using an offset throw line.

There are some other existing methods to gain access to the patient in these circumstances, including HEC operations with the use of a telescopic pole, “super longline” by the rescue helicopter service or “very long line” evacuations with static rope, where the longest reported rope length has been 1000 m in Romsdalen, Norway (July 2019). One possible advantage of using very long-line evacuations is the reduction of downwash from the rotor on the accident site. This could avoid air-filling of a parachute if the patient is still attached to it. Our study focuses on the offset technique, as it is a low-cost method which is realistic to implement in the current Norwegian system. Offset technique is considered as an additional rescue method to existing procedures, e.g. rescue procedures conducted by the rescue helicopter service.

## Method

A Safe Job Analysis, often referred to as an SJA, is a simple, qualitative risk analysis methodology used to identify hazards associated with a task to be performed [9, 10]. The method can be used on a wide range of work tasks. In this study, conducted during the last week of May 2021, the people who performed the tasks were included in the analysis [9]. The implementation of the analysis was led by a risk analyst. After completion of the tasks the participants were debriefed using a semi-structured interview focusing on the risk driving momentums. The interview was recorded and analysed.

The SJA was carried out by dividing the work assignments that was executed into tasks and sub-tasks. Hazards and conditions that could lead to dangerous situations were identified for each task/sub-task (Table 1).

**Table 1:** Output from the safe job analysis

Task step	Description	Subordinate task steps	Hazards	Possible consequences	Safety barriers	Changes in existing procedure: Proposals for risk-reducing barriers
1	Reconnaissance	Reconnaissance	Loose objects above the accident site Personnel moving above the accident site. Risk of falling rocks Length of static rope selected is too short	May pose a risk to the helicopter Rock falls can pose a danger to the helicopter. Can lead to personnel injury and material damage May cause climbers to be unable to drag the HEMS rescuer to the accident site (it will be too heavy)	Thorough reconnaissance during ROPE. If there is a rappelling rope down to the accident site or there are other loose objects above the accident site, the helicopter crew must make sure that they have been secured Thorough reconnaissance during ROPE. Make sure that there are no personnel above the accident site. Risk assessment of the mountain/terrain quality. Thorough reconnaissance during ROPE	Clarification in the procedure: during reconnaissance one must also assess whether any rappelling rope used by climbers to get to the accident site must be removed or secured before an offset technique operation *Other hazards and barriers to prevent them occurring are covered by existing procedure(s)
2	Leaving the rig site and arrival at the scene (approach)	The HCM attaches the offset throw line to the central loop of the climbing harness	The HCM forgets to attach the offset throw line in the central loop of the climbing harness	Time delay in mission. It will take longer before the rescuer can start preparing the patient for evacuation	Covered by existing checklist (SIST).	*Not found expedient to specify this in the procedure. Hazard identified is more of theoretical interest
		Last check, SIST, is carried out	The SIST check is not carried out or certain elements of the SIST are forgotten	Can contribute to additional consequences for the rescuer and patient, i.e., insufficient safety barriers	Procedure and training state that the SIST check shall be carried out. Experience shows that SIST is always carried out	Already implemented in the procedure
		The helicopter is positioned as close to the accident site as possible. The maximum throw length is approximately 15 m	The helicopter’s positioning is too far from the accident site. Throw length will be more than 15 m	Unable to access accident site	Thorough reconnaissance and assessment of throwing distance during ROPE. One should not try to gain access with a throw line if the throwing length exceeds 15 m	Already implemented in the procedure
		Hand signal “hold height” is given, and the HCM prepares the offset throw line. The ideal throwing position is 5 m above the accident site	Throwing position is more than 5 m above the accident site	May contribute to more failed throws. Longer time before the HCM can access the accident site. May result in the rescuer not arriving at the scene of the accident	Thorough reconnaissance and assessment of throwing distance during ROPE	Clarification in the procedure about why the ideal throwing position is 5 m above the accident site. Clarification could contribute to a better understanding of what is an ideal throwing position. More focus among the crew members regarding what is the ideal throwing position will then be achieved
		The offset throw line is thrown to the injured person who has received a brief in advance or to climbers involved. The climber holds the telescopic pole out to the side as an aiming point for the throw line	Personnel at the scene of the accident are not informed about what will happen when using an offset throw line Climber loses the telescopic pole Lost communication with the wireless intercom radio (Polycon MP50) The climber keeps the telescopic pole pointed at the HCM and not out to the side	Personnel not secured at the scene of the accident. Can lead to unprovoked fall injury Telescopic pole falls to the ground. Risk of material and/or personal injury Loss of communication between the HCMand the helicopter May contribute to more failed throws. Longer time before the HCM can access the scene of the accident. May result in the HCM not arriving at the scene of the accident	The helicopter crew shall have had telephone contact with personnel on the accident site before using the offset throw line. If there is no contact, the HCM can use the telescopic pole or wait until trained and briefed personnel have arrived at the accident site Telescopic pole shall be attached with a weak link to the rescuer or a fixed object Test of communication before the operation is started. Bring a back-up Polycon in the helicopter. Tetra handsets are carried by HCM Brief climbers before assignments. It is important to explain why the telescopic pole should be held out to the side as an aiming point for the throw line	Covered in current procedure Covered in current procedure Clarification in the procedure that Tetra radio must be carried by the HCM. This is particularly important, as lacking or incomplete communication between the crew members can be particularly critical with the offset technique, as the HCM is connected to the helicopter during the entire rescue mission and, in some situations, connected at the same time to both the mountain and the helicopter via the double attachment procedure Clarification in the procedure on whether the communication test must be carried out before the rescue operation is started Clarification in the procedure as to why the climbers should hold the telescopic pole aimed at the HCM and not out to the side. Clarification could contribute to a better understanding of this point for the crew members. More focus among the crew members regarding what is the ideal position for the telescopic pole will then be achieved and passed on to the climbers. Without clarification in the procedure, this point could fall out of the brief with climbers; the result could often be that the climbers tries to catch the rope and thus holds the telescopic pole straight towards the HCM. More difficult to establish contact with the HCM and the scene of the accident then
		HCM throws the line and misses. If the line misses on the first attempt, the line is coiled into bays and a new throw is made	Unable to throw offset throw line Offset throw line gets attached unintentionally to a fixed object	Unable to access accident site Helicopter accidentally attached to a fixed object	Thorough reconnaissance and assessment of throwing distance during ROPE. One should not try to gain access with an offset throw line if the throwing length exceeds 15 m Weak link at the end of the telescopic pole that breaks at 70 kg Thorough briefing of the personnel at the scene of the accident. Weak link that breaks at 70 kg. A knife or scissors must be available and ready at the accident site	Clarification of why the throw length should not exceed 15 m. A clarification in this area will contribute to more awareness of the recommendation in the procedure HCM must have access to two offset throw lines If one accidentally gets stuck and is cut with a knife or scissors, the rescuer will be able to gain access to the accident site with a second offset throw line
		Physician reports: “Rescuer throws the line”. “Rescuer hit/missed”	Lack of notification from the physician, or insufficient notification	The pilot has a different situational awareness from the rest of the helicopter crew. May cause injury to the HCM	Thorough brief of the offset technique before a mission. Also, clarification of how the physician reports to the pilot	Covered by existing procedure
		When a connection has been established, with the offset throw line between the HCM and climber, the climber pulls in the slack on the line and drags the rescuer to the scene of the accident. The hand signal “down” is used by the rescuer to position the helicopter down. A calm descent is of importance	Personnel at the accident site lose the offset throw line Excessive load on the offset throw line. Weak link breaks Personnel at the accident site are unable to drag the HCM to the scene of the accident The rotor disk can hit the ropes that climbers has used to rappel down to the accident site. This can happen in situations where the rappel ropes stand out from the rock wall due to an overhanging rock wall	Loss of contact with the accident site Loss of contact with the accident site HCM unable to access accident site Helicopter crash	There must have been radio contact with personnel at the scene of the accident before the offset technique is initiated. A plan is briefed for what to do if the HCM misses with the throw line ◊ HCM coils up the throw line and throws again HCM physically holds his hand on the knot where the weak link is. This will reduce the physical load on the weak link Do not descend with the helicopter too quickly so that the burden on personnel at the accident site becomes too great. Strive for the HCM to be a little over height when he is guided to the scene of the accident Thorough reconnaissance of climbers rappel ropes and an assessment of whether they should be removed before the approach and offset technique are initiated	Covered by existing procedure Covered by existing procedure Covered by existing procedure Clarification in the procedure about the importance of assessing whether the climbers rappel ropes should be removed before approaching the patient in the reconnaissance phase
3	Preparation of the patient	The HCM evacuates the patient in their own harness, or climbers must have prepared the patient according to written procedures	HCM detaches from the static rope	Loss of contact with the helicopter and the possibility of evacuating from the scene with the helicopter	A thorough brief and a procedure which states that the HCM must not disconnect from the static rope	Covered by existing procedure
		Topography and rock formations at insertion points determine whether there is a need to use the double attachment procedure	The helicopter is unable to maintain a steady hover while the HCM is connected to a fixed object during the double attachment procedure	The consequences can be critical for the HCM but can also be insignificant. The consequences depend on whether the helicopter descends or ascends	Thorough reconnaissance and assessment of the length of the static rope before the mission. Test of communication equipment. Tetra radio is brought along by the HCM	Covered by existing procedure
		The HCM either leaves the offset throw line at the scene of the accident or packs the line in the throw line bag	–	–	–	–
4	Leaving the scene and evacuation, underslung, by the helicopter to the rig site (departure)	The HCM uses the “Positioning Sign” to position the helicopter if necessary. The static rope must be tight, to avoid snagging of the rescuer and patient, approximately 0.5 m slack	–	–	–	–
		If a double attachment must be used on patients or climbers, this is done in accordance with procedure	Unintentional double attachment of the helicopter	Personnel or material damage	Thorough brief of double attachment procedure before mission. Annual recurrency training	Covered by existing procedure(s).
		HCM performs “SIST” check	–	–	–	Covered by existing procedure.
		HCM signals “up” when everything is ready. Evacuation from the scene of the accident can be planned and carried out in accordance with two options: 1. The HCM and patient carry out a controlled pendulum from the scene of the accident. The physician reports “Rescuer clear of the ground” as they leave the scene of the accident. This option is recommended with a triangle harness or when using the patient’s own harness. 2. The climber who is left at the scene uses a short tag line with a free-running Munter hitch for a controlled extrication. This is to reduce the pendulum movement. Recommended when using a rescue stretcher.	Helicopter ascends too fast. Too much slack in the static rope and a fall from the accident site. Departure from the scene of the accident is done with the help of a climber, but where climber does not use a thigh short tag line	HCM and patient depart unintentionally from the accident site. Contact with terrain or obstacles. Static fall in the static rope. Uncontrolled use of the short tag line, which can result in consequences for both the rescuer and the patient.	Thorough reconnaissance during ROPE and briefing of procedure. Aim for a maximum of 0.5 m of slack on the static rope. AAK Safety has carried out tests which show that there is no need for additional fall arresters at the static rope as there are enough dynamics in the rope system as of today. Thorough brief on the offset procedure to climbers before missions and explanation of why the short tag line is used.	Clarification in procedure regarding why a slow ascent. Clarification that one should aim for a maximum of 0.5 m of slack on the static rope. This is to prevent the HCM from being inadvertently lifted out of the scene of injury or a static fall in the static rope. Covered by existing procedure.
5	Evacuation of climber(s)	There must be a plan for the retrieval of climbers, including a helicopter-independent plan.	*Same as when evacuating a patient with the offset technique	*Same as when evacuating a patient with the offset technique	*Same as when evacuating a patient with the offset technique	–
		If the patient’s condition is not time-critical, then the climbers can be evacuated using the offset technique. The physician on duty assesses this based on the patient’s condition.				
		If the patient’s condition is critical/urgent, treatment and transport have the highest priority. Climbers moves to the same point they were inserted, waiting to be picked up at a later time. Alternatively, a rappel out of the wall				

Our study focuses on analysing the offset static rope evacuation procedure. A technical rescue procedure has been developed to describe how a rescue operation using the offset technique should be carried out. The procedure is an adjusted variant of a similar technique used by rescue personnel in Yosemite National Park, USA [11]. Prior to the procedure, an internal company risk analysis was carried out and quality assured by the company’s Safety Action Group. We identified a need for a more systematic risk analysis of the offset rescue technique, bringing in more experience from crew members, in order to obtain better insight into the potential hazards of using the offset rescue technique. The output from the analysis is further used to evaluate the existing procedure for the offset technique, to see the extent to which different identified hazards are eliminated or considered, in the way the procedure is described.

**Fig. 2:**
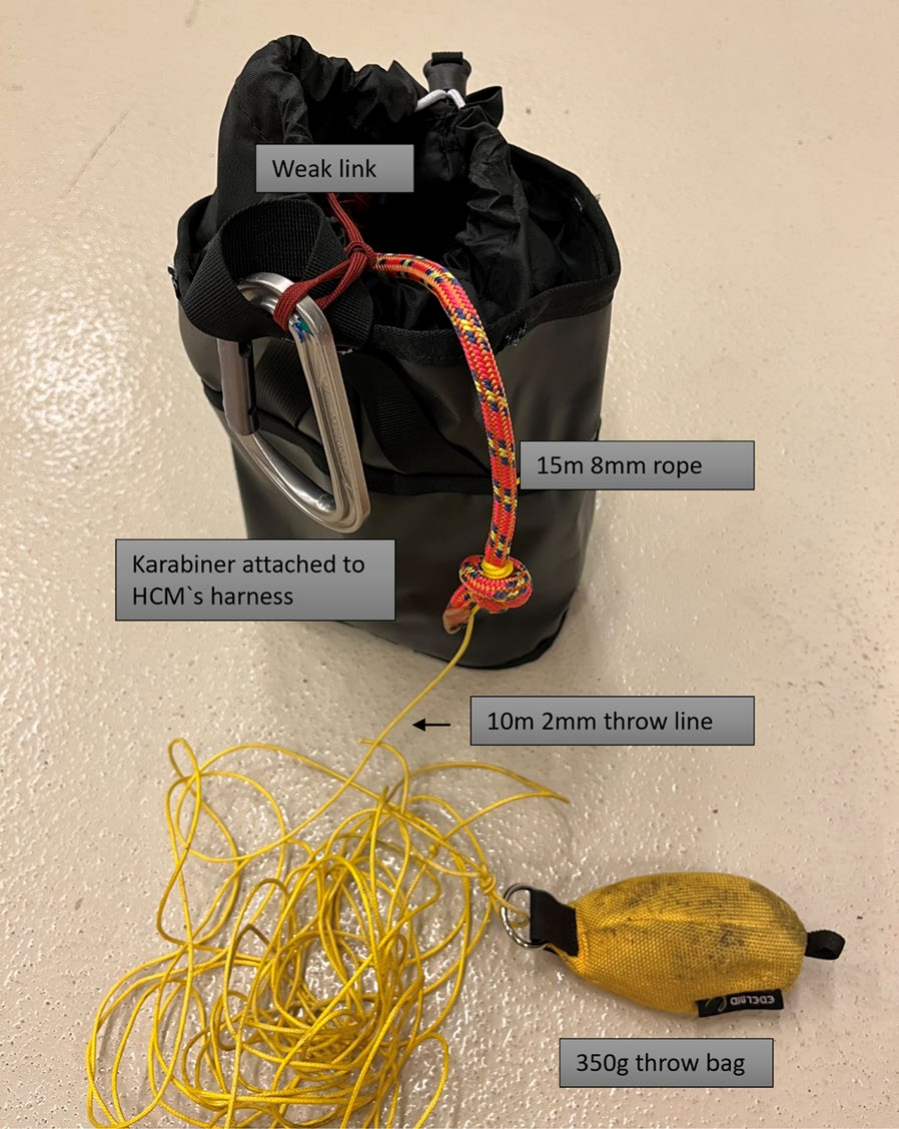
Offset throw line with weak link. Photo: Håvard Mattingsdal

In missions requiring the offset technique, the HCM can establish a connection to the patient by using an offset throw line. One part of the offset throw line is connected to the HCM’s harness, while the other part is thrown by the HCM and caught either by personnel at the accident site or in cooperation with volunteer rescue climbers. The offset throw line is equipped with a weak link, as shown in Fig. 2. The weak link is essentially a link designed to ensure that an emergency release breaking point is included in the chain. The weak link is attached to the HCM`s harness and breaks at a load of 70 kg in case of an unintentional entanglement of the throw line [12].

**Fig. 3:**
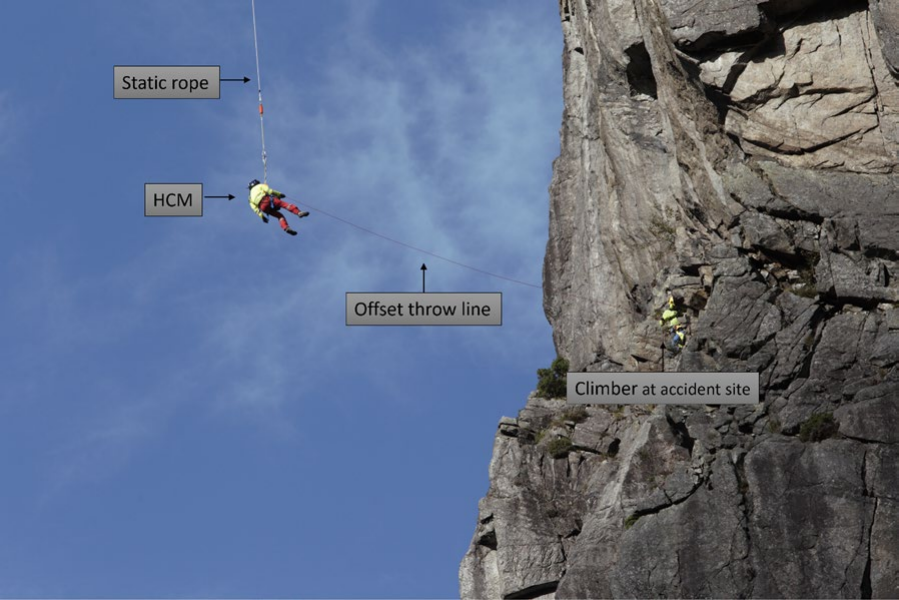
Climber at the accident site receives the throw line and drags in the HCM. Photo: Hugo Bergsaker

With the help of the offset throw line, the climber can, in coordination with the helicopter’s descent, drag the HCM to the accident site; see Fig. 3.

The HCM can also gain access to the accident scene without a climber. In such situations, the offset throw line will be thrown to the patient or to a nearby person, who can drag the rescuer to the scene. In the latter situation, the personnel at the scene must understand the rescue operation and there must have been telephone contact with the on-site personnel.

Unlike most traditional static rope evacuations, in offset rescue operations, the HCM cannot disconnect from the static rope during the preparation of the patient. If a disconnection occurs, no physical contact can be made between the helicopter and the HCM, as the helicopter must be out of plumb from the scene, due to the particular conditions of the terrain. Patients requiring offset technique can often be in complex terrain where there might be a need for the double attachment procedure.

**Fig. 4:**
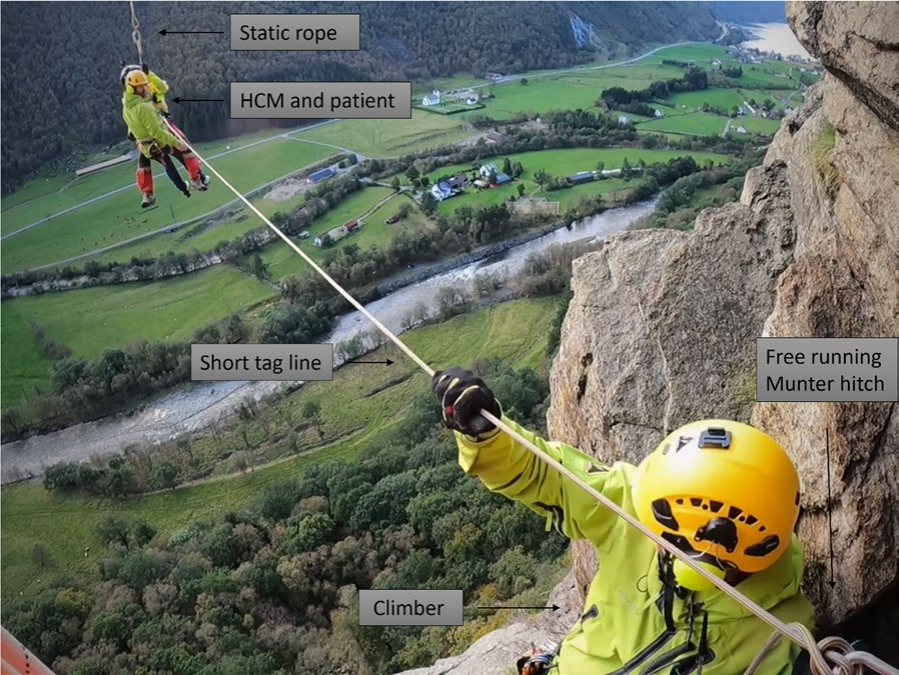
Climber steers the HCM and patient out of the scene with a short tag line controlled with a free-running Munter hitch. Photo: William Ottestad

As the helicopter is out of plumb, the HCM and patient can either be evacuated from the scene with a controlled pendulum, or the climber can steer the rescuer and the patient out of the scene using a short tag line controlled with a free-running Munter hitch, as shown in Fig. 4.

The hand signal “free of obstacles” is given by the HCM when the short tag line is free from the scene. The HCM and patient can then be evacuated, underslung, by the helicopter to the rig site, where further treatment can be provided by the emergency physician if necessary. The final step of the rescue operation will remain the same as for all other rescue operations carried out by HEMS.

*Ethics*

Voluntary consent was obtained from all crew members before proceeding with data collection from all the potentially involved participants in the study. The study was approved by the Norwegian air ambulance data collection officer, in accordance with the rules from the Norwegian Social Science Data Services.

## Results

An overview of the outputs from the SJA is given in Table 1. In the SJA, we systematically review the offset rescue technique by dividing the work into tasks and reviewing each task, to assess the hazards associated with it. In Table 1, the offset rescue operation is split into the following five sub-tasks:

1. Reconnaissance
2. Leaving the rig site and arrival at the scene
3. Preparation of patient
4. Leaving the scene and evacuation, underslung, by the helicopter to the rig site
5. Retrieval of climber(s)

These sub-tasks are the same tasks highlighted in the procedure for the offset technique.

The results from the SJA are based on feedback from five pilots, four HCMs and five emergency physicians. The crew member composition includes highly experienced to less experienced crew members. The dialogue with the different crew members was organized by the authors, consisting of a professor in risk management together with a subject-matter expert HCM and an emergency physician.

Before the dialogue with the different crew members on potential hazards for the different steps of the offset technique, we organized some training sessions related to static rope evacuation. A full training session included (1) a traditional static rope rescue operation with a 30-m and a 60-m rope, (2) an offset rescue technique with a 30-m and a 60-m rope, and (3) an offset rescue technique in really steep terrain, using a 60-m rope. All the training scenarios with the use of the offset technique were conducted in complex terrain, requiring the double attachment procedure [8]. All training missions were conducted in accordance with approved procedures, limitations and risk analysis for static rope rescue missions.

Some crew members did all five training runs, but some only participated in some of the runs. All the crew members that gave input to the SJA participated in at least one of the offset training runs.

In the risk analysis, we focus only on potential hazards associated with the offset rescue technique. Attention is not given to the risks of the different hazards identified. The reason for this must be seen in relation to the aim of our study, whose focus is on the procedure for the offset rescue technique and an evaluation of the same. We then need to gain insight into the extent to which different identified hazards are eliminated or considered, in the way the procedure is described. The classification and categorizations of risks for the identified hazards are then not relevant, given the main aim of our study.

It can be pointed out that hazards which are solely of theoretical interest are omitted from the analysis. This is to avoid making changes to the existing procedure to avoid hazards that are solely of theoretical interest. All the results are shown in Table 1. The main findings from the analysis are given below and categorized for each task of the offset rescue operation.

*Sub-task 1: reconnaissance*

In the reconnaissance phase, a choice is made as to how the rescue operation should be executed. When deciding upon evacuation by the use of a static rope, one must decide on the length of the rope to be used—one which provides the pilot with good visual references—and, further, on the type of evacuation procedure to be used—the traditional one or the offset technique. Based on the SJA, the choice of rope length has been identified as being more critical when using the offset technique than when using the traditional underlying rescue operation. Two main arguments for this were identified. In an offset rescue operation, a too short rope may contribute to the HCM not reaching the scene. This may happen as the rescuer cannot make ground contact given the helicopter’s offset location. There may also be situations where the length of the rope is sufficient in itself but where the HCM still cannot get to the scene, as dragging him/her to the scene proves too heavy for the climber. To make the work for the climber less heavy, the flight height must be reduced, which is not necessarily possible due to the terrain. This was identified as a problem in one of the training runs.

*Sub-task 2: leaving the rig site and arrival at the scene*

Compared to the traditional static rope evacuation, it is the arrival at the scene that is different when using the offset rescue technique. During the traditional static rope evacuation, the HEMS crew can arrive at the scene directly. When using the offset technique, you need help from a person at the scene to establish this contact. This can be done with help from volunteer rescue climbers, a person close to the patient or the patient him/herself. In this step of the offset technique, several hazards may occur, as shown in Table 1. Most hazards will just lead to more time being spent before reaching the scene and thereby more time spent before evacuation of the patient can start. But, without the offset technique, one could not gain access to the patient from the helicopter.

From Table 1, we see that there are some situations that may arise during offset operations that affect the safety of the crew members. First, one safety issue which might occur is if the HCM does not complete a final check of his/her personal protection equipment, i.e., harness, attachment to static rope or offset throw line. This hazard is very unlikely to occur, due to the established checklists, safety barriers and routines before a rescue mission is executed. Second, there is a risk of the helicopter being positioned too close to the terrain, which might pose a serious hazard for the whole helicopter crew. During a rescue mission in cooperation with climbers, rappel ropes might also pose an additional hazard for the helicopter. Third, there is a risk of the offset throw line accidentally becoming entangled with a fixed object at the accident site. The weak link on the throw line is designed as a safety barrier if this should occur.

*Sub-task 3: preparation of patient*

In the offset technique, the helicopter and the HCM may lose contact with the scene. This will happen if the rescuer disconnects from the helicopter during the offset operation. This should not be done, as is clearly described and specified in the procedure for the offset technique. This aspect should also be highlighted in the briefing between the crew members before an offset rescue operation starts.

In addition to this, by using the offset technique, one will sometimes end up in a situation where the HCM is connected to both the helicopter and to the scene at the same time, due to complex terrain and use of the double attachment procedure. Critical situations may then arise if the helicopter is not able to maintain a steady hover. This might also be a hazard in a traditional static rope evacuation when using the double attachment procedure. Should this emergency occur, the helicopter has the possibility to disconnect the rope from the helicopter, hence allowing for a fly away. Disconnecting the rope from the helicopter represents a serious hazard for the HCM hanging at the end of the rope and is only to be used in an emergency as a last option.

*Sub-task 4: leaving the scene and evacuation, underslung, by the helicopter to the rig site*

Using the offset technique, the HCM and patient can leave the scene autonomously using a controlled pendulum. If using the short tag line when leaving the scene, there is a need for a climber to control the free-running Munter hitch.

In cooperation with climbers, it is important that the climbers are thoroughly briefed on the offset technique in advance. In one of the training runs, where a controlled pendulum was not possible due to obstacles in the evacuation axis, a situation was identified where the climber did not follow the offset procedure: he/she did not control the Munter hitch on the short tag line when the HCM was leaving the scene, allowing for a pendulum. There can be several reasons why one does not follow the procedure. One reason could be that the climber was not thoroughly briefed in advance. Another reason is that the extent of the job task itself is complex, which contributes to difficulties in following the procedure as intended. A third reason is that one does not fully understand what was conveyed in the briefing. The procedure in itself is not difficult, but we discover that it can be fruitful to explain during the briefing not only what to do but why things should be done as described in the procedure. A fourth and probably most likely reason is that most climbers are not used to operating in a helicopter rescue environment. This has been observed in several similar situations and might have affected the overall stress level of the climber, allowing for an adverse event to happen.

During the risk analysis workshop, it was highlighted that the use of the short tag line is of special importance if obstacles are present when leaving the scene and in the evacuation axis (e.g., a canyon/gully), underslung, by the helicopter to the rig site. Without the use of the short tag line, there will be a risk of a strong pendulum movement when the HCM and the patient leave the scene, which may have an impact on the safety of both the rescuer and the patient, due to the terrain.

*Sub-task 5: retrieval of climber(s)*

After leaving the scene and evacuation, underslung, by the helicopter to the rig site, one needs to retrieve the climber(s). This can be done by using the offset technique or by the climbers themselves. What happens after having flown out of the scene with a patient will not have an impact on the offset procedure. All hazards that may occur during a possible retrieval of the climber(s) will be taken care of by conditions that are covered in the sub-task ‘Leaving the scene and evacuation, underslung, by the helicopter to the rig site’.

## Discussions

### The utility of using an SJA in the present study

Alternative methods to the SJA could also be used. Given the purpose of our analysis, we could, for example, also use a coarse risk analysis or a hierarchical task analysis. The analysis, as carried out in this study, would have been performed similarly if one had chosen a coarse risk analysis or a hierarchical task analysis. The job that is to be executed (the offset rescue technique) will, regardless of method, be divided into sub-tasks. How the risk is described for the various hazards identified for all the sub-tasks identified will, for each method, largely depend on the analyst. In our analysis, we do not analyse the risk for the different hazards identified, as this aspect is not considered important, given the purpose of our analysis.

Although we could perform our analysis by using another risk analysis method, we decided to use an SJA. There are two main reasons for this. Firstly, our focus is on the offset technique, which is a specific job that is to be executed. This is the basis for an SJA, which is also the basis for a hierarchical task analysis. We decided to use an SJA as, compared with the hierarchical task analysis, it is freer in terms of the steps that are to be included in the analysis. The hierarchical task analysis is often carried out, together with a Sherpa, as a basis for assessing risk. Based on the purpose of our analysis, there is less need to carry out a Sherpa and less need to carry out all the steps required when adopting a hierarchical task analysis. The focus in our analysis is solely on the identification of hazards associated with the offset rescue technique that is to be executed.

The SJA is a well-known risk analysis method. It is used in many different industries, and numerous studies exist in which the SJA is used to develop procedures to ensure the safe planning/execution of different jobs [9]. It can also be used for the further development of existing procedures for how different jobs should be performed [10].

### Is there a need to modify the procedure for the offset technique?

The risk analysis workshop and the SJA’s main objective was to identify potential hazards for helicopter rescue operations using the static rope offset technique and, thereby, to improve the procedure for such operations. This may lead to improved safety for both patients and crew members during offset rescue operations. Our study identified three important factors in the existing offset technique procedure:

A. Clarifications in the procedure, focusing not only on what is to be done but also why.
B. A need for two offset throw lines.
C. The importance of communication and a brief with the climbers.

### Is evacuation by the offset technique more dangerous than traditional static rope evacuation?

The aim of the study is not to compare the risk of the offset technique with that of the traditional fixed rope evacuation technique. This is, however, an interesting aspect that is important to focus on in future studies. A preliminary study has already been initiated, in which a special focus is on the difference in perceived workload for the various crew members when comparing the offset technique with the traditional fixed rope evacuation technique.

Based on the experiences with training in the offset technique and on interviews with different crews, we have gained some insights. We have seen the importance of having a clear briefing before using the offset technique. A clear briefing between the different crew members, as well as between the crew members and the climber(s), is considered important. With no clear briefing, evacuation by the offset technique may not go as intended. This is the reason why we consider it important to clarify why some aspects in the procedure are as they are and to not only focus on how evacuation by the offset technique should be performed. In this way, one will increase awareness of the different elements within the procedure. This could in turn lead to clearer communication between the crew members and the climber(s). This was also reported by the participants during the debriefing sessions.

### No focus on risk for the identified hazards in the SJA

In our analysis, we focus on what can go wrong and how to control it. This information is needed to decide whether the current procedure for the offset technique is appropriate to use. Assessment of risk for the identified hazards is not of importance, given the purpose of our analysis. However, an overall assessment of risk is made for each identified hazard. This is to avoid changes and adjustments being made in the existing procedure for the offset technique to accommodate hazards that are merely of theoretical interest. Such an aspect has also been included in other risk analysis methods. The HFMEA (Healthcare Failure Mode and Effect Analysis) method, for example, includes one question in the decision tree prior to the decision on investments in a safety measure (“Is the hazard so obvious and readily apparent that a control measure is not warranted?”) [13–15].

It can be noted that if, in our SJA, we had to assess the risk for each of the identified hazards, we would not have done this solely by focusing on probabilities and consequences, which is often done. Such a focus ignores other important aspects that need to be taken into consideration when assessing risk, such as uncertainty and strength of knowledge. Emphasis on strength of knowledge and uncertainties is something that has been given much attention for many years. The importance of taking these aspects into consideration when assessing risk in an SJA is particularly highlighted in Aven (2015) [9].

## Conclusions

Based on this study, we consider that the procedure for the offset rescue technique is safe and appropriate, but that there is a need for some adjustments. In this paper, we highlight the importance of clarifying ‘why’ for some of the aspects in the procedure, and not only focusing on what to do. Experiences with training on the offset technique, in addition to interviews with several crew members, show that information on ‘why’ may lead to better communication between the crew members and between the crew members and the climber(s) during their briefing on what to do before offset evacuation. Potential hazards may then be avoided. We also recommend that the HCM has access to two throw lines and regularly trains on the manual skills of throwing the offset line. The present work has contributed to changes in the existing procedure for implementing the offset rescue technique.

## Data Availability

Not applicable.
